# Polarized expression of the membrane ASP protein derived from HIV-1 antisense transcription in T cells

**DOI:** 10.1186/1742-4690-8-74

**Published:** 2011-09-19

**Authors:** Isabelle Clerc, Sylvain Laverdure, Cynthia Torresilla, Sébastien Landry, Sophie Borel, Amandine Vargas, Charlotte Arpin-André, Bernard Gay, Laurence Briant, Antoine Gross, Benoît Barbeau, Jean-Michel Mesnard

**Affiliations:** 1 Université Montpellier 1, Centre d'études d'agents Pathogènes et Biotechnologies pour la Santé (CPBS), Montpellier, France; 2 CNRS, UM5236, CPBS, F-34965 Montpellier, France; 3 Université Montpellier 2, CPBS, F-34095 Montpellier, France; 4 Université du Québec à Montréal, Département des sciences biologiques and Centre de recherche BioMed, Montréal, Canada; 5 Salk Institute for Biological Studies, San Diego, USA

## Abstract

**Background:**

Retroviral gene expression generally depends on a full-length transcript that initiates in the 5' LTR, which is either left unspliced or alternatively spliced. We and others have demonstrated the existence of antisense transcription initiating in the 3' LTR in human lymphotropic retroviruses, including HTLV-1, HTLV-2, and HIV-1. Such transcripts have been postulated to encode antisense proteins important for the establishment of viral infections. The antisense strand of the HIV-1 proviral DNA contains an ORF termed *asp*, coding for a highly hydrophobic protein. However, although anti-ASP antibodies have been described to be present in HIV-1-infected patients, its *in vivo *expression requires further support. The objective of this present study was to clearly demonstrate that ASP is effectively expressed in infected T cells and to provide a better characterization of its subcellular localization.

**Results:**

We first investigated the subcellular localization of ASP by transfecting Jurkat T cells with vectors expressing ASP tagged with the Flag epitope to its N-terminus. Using immunofluorescence microscopy, we found that ASP localized to the plasma membrane in transfected Jurkat T cells, but with different staining patterns. In addition to an entire distribution to the plasma membrane, ASP showed an asymmetric localization and could also be detected in membrane connections between two cells. We then infected Jurkat T cells with NL4.3 virus coding for ASP tagged with the Flag epitope at its C-terminal end. By this approach, we were capable of showing that ASP is effectively expressed from the HIV-1 3' LTR in infected T cells, with an asymmetric localization of the viral protein at the plasma membrane.

**Conclusion:**

These results demonstrate for the first time that ASP can be detected when expressed from full-length HIV-1 proviral DNA and that its localization is consistent with Jurkat T cells overexpressing ASP.

## Background

Human lymphotropic retroviruses, such as human T-cell leukemia virus type 1 (HTLV-1) or human immunodeficiency virus type 1 (HIV-1), have evolved multiple strategies to direct the synthesis of a complex proteome from a small genome, which involves alternative splicing, internal ribosomal entry sites, ribosomal frameshifting, and leaky scanning [1]. Retroviral genomes are transcribed through a proviral DNA intermediate integrated into the cell chromosome and expressed by the host transcription machinery. All retroviral genes have been thought to be transcribed through a single promoter located in the 5' long terminal repeat (LTR) of the provirus. However, early studies have described the presence of conserved open reading frames (ORF) in the complementary strand of the HIV-1 and HTLV-1 proviruses, suggesting the existence of viral mRNAs of negative polarity produced from the 3' LTR [2,3]. More recently, we and others have conclusively demonstrated the presence of such antisense RNAs in cells infected with HIV-1 or HTLV-1 [4-7].

In the case of HTLV-1, the antisense strand-encoded protein that we have termed HBZ for HTLV-1 bZIP factor [8] is a c-Fos-like nuclear factor [9,10] that attenuates the activation of AP-1 [11-14] and down-regulates viral transcription [15,16]. *In vivo *studies using a rabbit model have shown that HBZ is involved in the establishment of chronic viral infections [17], indicating that HBZ could play a key role in the escape of HTLV-1 from the immune system by controlling viral expression [18,19]. Interestingly, we have recently demonstrated that HTLV-2 encodes an antisense protein (called APH-2 for antisense protein of HTLV-2) that also represses viral transcription [20].

**Figure 1:**
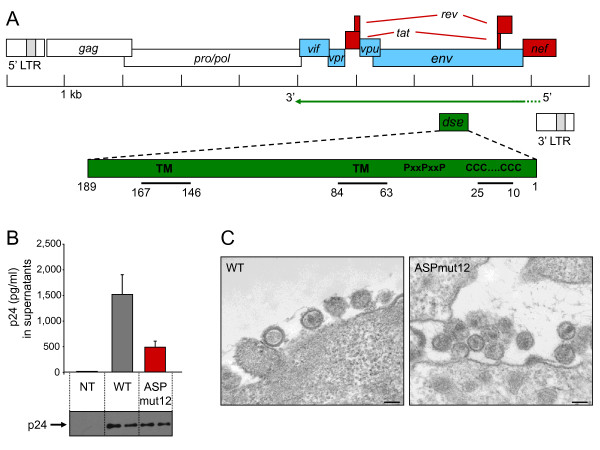
**Characterization of the HIV-1 ASP mutant proviral clone**. (A) Schematic representation of the HIV-1 proviral genome. The viral ORFs are presented based on the nature of their encoding transcripts, i.e. multiply-spliced, mono-spliced, and unspliced sense transcripts (red, blue, and white). The antisense strand-encoded *asp *ORF (green) is also indicated. The reported *asp *coding region is further indicated below showing the two cysteine triplets, the SH3 binding motif (PxxPxxP), and potential transmembrane regions (TM). The numbers shown indicate amino acid positions. (B) Reduction of extracellular p24 Gag levels from 293T cells transfected with ASP-deficient HIV-1 proviral DNA. 293T cells were cotransfected with pNL4.3WT or pNL4.3ASPmut12 and pRcActin-LacZ. Forty-eight hours after transfection, supernatants were harvested and quantified through a p24 ELISA assay. Results are presented as the average p24 value +/- S.D. of β-galactosidase-normalized values from three independently transfected cell samples. Cell lysates were prepared from non-transfected 293T cells (NT) or cells transfected with pNL4.3WT or pNL4.3ASPmut12. Western blot analyses (under the histogram) were conducted on these preparations using anti-p24 and HRP-conjugated goat anti-rabbit IgG antibodies (two independent transfections are presented per condition). (C) Analysis of WT and ASPmut12 virion morphology. Virus particles produced from 293T cells transfected with pNL4.3WT or pNL4.3ASPmut12 were analyzed in thin-layer electron microscopy. The black bars correspond to a scale of 100 nm.

Although all functional HIV-1 genes are thought to be transcribed from the sense proviral DNA strand only, a very recent study has shown that cryptic epitopes derived from an HIV-1 antisense ORF are generated in infected CD4+ T lymphocytes [21], confirming the production of viral proteins from antisense transcription. Among the different negative sense ORFs found in HIV-1 [2,6], the *asp *(for antisense protein [22]) ORF, encoded by the complementary strand to the gp120/gp41 junction of the *env *gene (Figure 1A), is the most conserved and the longest. Moreover, its presumed ATG initiation codon is also very well preserved. In addition, its position from the 3' LTR is extremely similar to the *hbz *ORF in HTLV-1 and the *aph-2 *ORF in HTLV-2. *Asp *codes for a highly hydrophobic protein [2] (Figure 1A) that has been found associated with virions released from infected cells [22]. Moreover, the ASP protein has been described to be recognized by antibodies present in patients infected by HIV-1 [23]. Here, we demonstrate for the first time that ASP is expressed in Jurkat T cells infected with a proviral clone, with an asymmetric localization of the viral protein at the plasma membrane.

## Results

### Construction and characterization of an HIV-1 ASP mutant proviral clone

**Figure 2:**
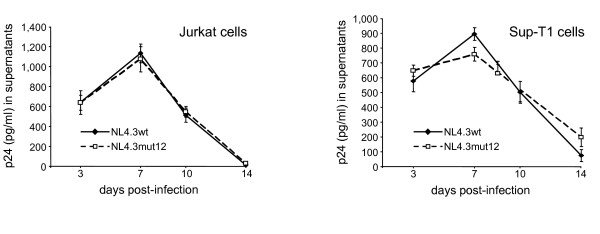
**HIV-1 infection of T cells is not affected by the absence of ASP**. HIV-1 viral particles harvested from 293T cells transfected with pNL4.3WT or pNL4.3ASPmut12 were used to infect Jurkat (A) and Sup-T1 (B) cells. Extracellular p24 levels were quantified on supernatant from triplicate infected samples and are presented as the mean value +/- S.D.

In order to study ASP, we first generated a mutated proviral clone in which a stop codon was inserted in frame to the *asp *ORF. This mutation resulted in termination of ASP at amino acid 12 of the published sequence [2] without altering the amino acid composition of the Env protein encoded on the sense strand. This resulting pNL4.3ASPmut12 construct was next transfected in 293T cells and compared for p24 production to 293T cells transfected with wild type (WT) pNL4.3. Transfected cells showing comparable transfection efficiency were then selected to evaluate their levels of extracellular viral capsid proteins. Interestingly, 293T cells transfected with the mutated proviral DNA showed lower extracellular p24 levels when compared to results obtained with the parental wild-type proviral DNA (Figure 1B). To determine whether p24 expression was also reduced intracellularly, cell lysates from transfected cells were analysed by Western blotting. As shown in Figure 1B, intracellular p24 levels were not affected by the ASP mutation. These data were confirmed in three different experiments, and analyses of p24 signals by densitometry further demonstrated equivalent p24 levels in cells transfected with the two tested NL4.3 proviral DNA (see the additional file 1, figure S1). Western blot analyses were also performed on the same preparation by using human anti-HIV-1 serum and confirmed that intracellular levels of viral proteins were not affected (data not shown). The effect of the mutation was also investigated on the structure of the viral particle by electron microscopy analysis, and normal-sized mature virions were found in preparations of WT and ASPmut12 particles (Figure 1C). The presence of unambiguous cone-shaped nucleoids was also observed in WT and ASPmut12 viruses. In addition, when Jurkat and Sup-T1 cells were infected with WT or ASPmut12 viruses, no significant differences in the levels of extracellular p24 were detected at different times post-infection between both viruses (Figure 2).

### The HIV-1 ASP protein localizes to the plasma membrane

To better characterize the ASP protein, its expression and localization were analyzed in Jurkat cells. Analysis of its amino acid sequence reveals a highly hydrophobic protein. Hydropathy and immunogenicity plots demonstrate a minimal number of soluble regions and suggest two transmembrane domains extending from amino acid 63 to 84 and amino acid 146-167 (Figure 1A). In its N-terminal region, the ASP sequence also revealed the presence of two conserved cysteine triplets (with a potential palmitoylation site for the first one) and two SH3-binding motifs with a typical proline rich sequence with a PxxP minimal core (Figure 1A). We thus presumed that the presence of potential transmembrane domains could lead to membrane localization of the protein.

**Figure 3:**
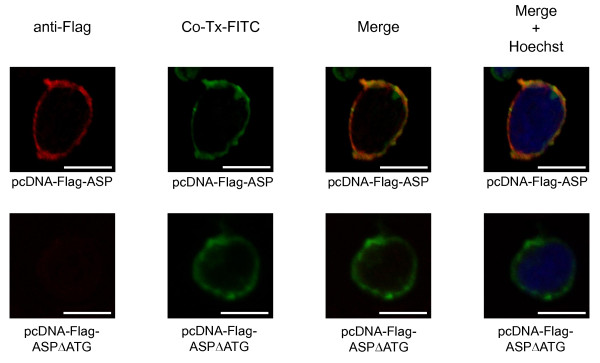
**HIV-1 ASP localizes to the membrane**. Jurkat cells were transfected with pcDNA-Flag-ASP expressing ASP tagged with the Flag epitope to its N-terminal end or with pcDNA-Flag-ASPΔATG. Localization of ASP to the membrane was visualized by confocal microscopy using FITC-Co-Tx and immunostaining with a primary anti-Flag antibody, followed by a secondary antibody coupled to Alexa Fluor 568. Nuclei were labelled with Hoechst. White bars correspond to a scale of 10 μm.

To test this hypothesis, Jurkat T cells were transfected with an ASP expression vector in which ASP was tagged with the Flag epitope at its N-terminal end. The transfected cells were co-stained with both FITC-Co-Tx (which binds to GM1 ganglioside, a component of the cell plasma membrane) and anti-Flag antibody and subsequently analysed on a confocal microscope (Figure 3). Image merging of both fluorescent signals confirmed the localization of ASP to the plasma membrane. The same approach was performed with cells transfected with the pcDNA-Flag-ASPΔATG expression vector, in which the initiation codon of Flag-ASP was replaced by a stop codon. No specific fluorescent signal was detected with the anti-Flag antibody as illustrated in Figure 3.

### ASP localizes differently at the membrane of Jurkat cells

**Figure 4:**
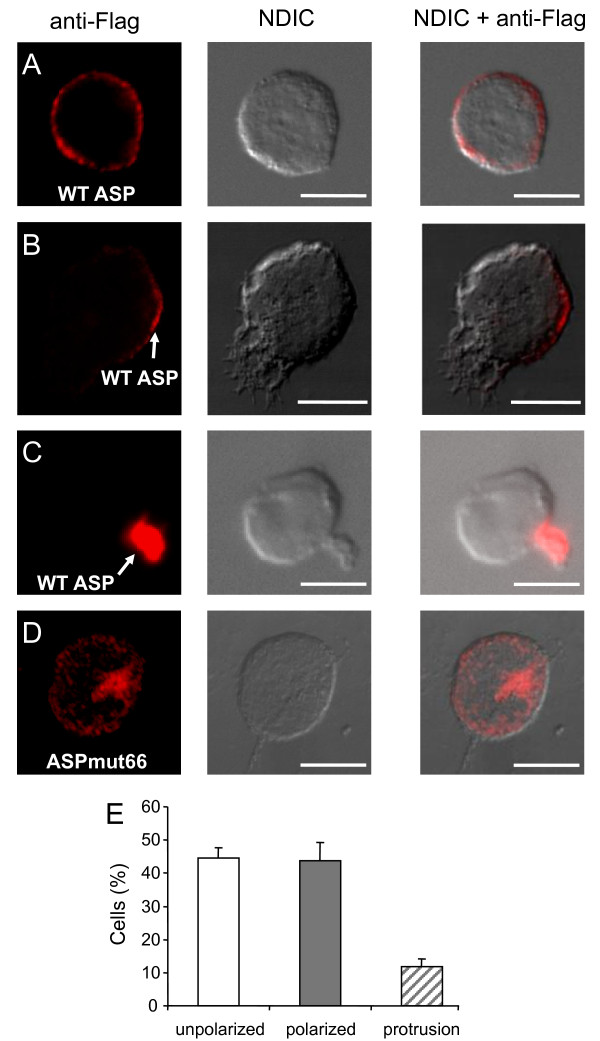
**Cellular localization of WT ASP and ASP-mut66 in transfected Jurkat T cells**. Jurkat cells transfected with pcDNA-Flag-ASP (A-C) or pcDNA-Flag-ASPmut66 (D) were layered on glass slides, fixed, permeabilized, and stained with fluorescence-labelled antibodies as described in Figure. 3. The morphology of the cell was assessed by Normaski differential interference contrast (NDIC). White bars correspond to a scale of 10 μm. (E) Percentage of the total transfected cells with ASP showing an unpolarized distribution (white bar), a polarized location (grey bar), or a localization into membrane protrusion (hashed bar). A total of 206 cells from three separate experiments were scored.

Although our results showed that ASP localized to the plasma membrane, two distinct sites were observed. In addition to its unpolarized localization to the plasma membrane (Figure 4A), ASP also showed an asymmetric distribution in ASP-expressing Jurkat cells (Figure 4B). Polarization of ASP to the plasma membrane was found in 44% ± 5 of transfected cells while unpolarized distribution corresponded to 44% ± 3 (Figure 4E). Moreover, ASP occasionally presented a strong localization into membrane protrusion (Figure 4C) corresponding to 12% ± 2 of transfected cells (Figure 4E). Such staining patterns were not observed in Jurkat cells tranfected with the negative control pcDNA-Flag-ASPΔATG (data not shown). We also analyzed the subcellular localization of an ASP mutant, called ASPmut66, corresponding to the first 65 amino acid residues of ASP, which were thus devoid of both potential transmembrane domains. Compared to the wild type, this mutant showed a different staining profile since ASPmut66 was not localized to the plasma membrane (Figure 4D).

**Figure 5:**
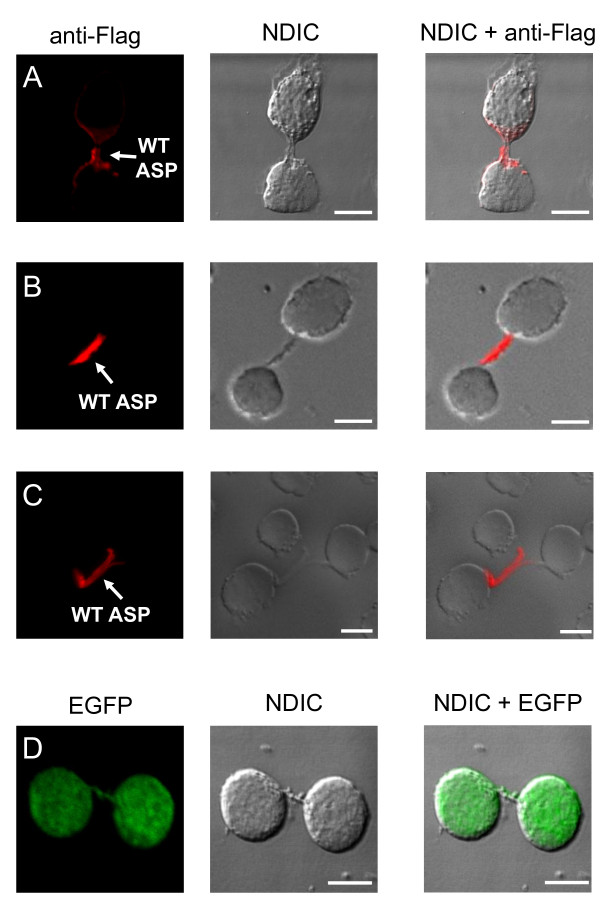
**ASP localization in membrane connection**. Jurkat cells transfected with pcDNA-Flag-ASP (A-C) or pEGFP (D) were layered on glass slides (A) or seeded at high density on polylysine-covered glass slides (B-D). The localization of ASP was analyzed as described above. The morphology of the cell was assessed by NDIC. White bars correspond to a scale of 10 μm.

Using this approach, we also detected ASP in membrane connections between two cells as shown in Figure 5A. Based on these results, we further analyzed the distribution of ASP in transfected Jurkat cells seeded on polylysine-covered glass slides at high density, thus favouring cell-to-cell interactions. Interestingly, an intense staining of ASP was found in membrane projections (Figure 5B). Moreover, the ASP staining can highlight a thin and long connection between neighbouring cells (Figure 5C). When similar analyses were performed in Jurkat cells transfected with pEGFP, the fluorescent signal demonstrated a diffuse pattern present not only in intercellular connections, but also in all cellular compartments (Figure 5D). The Scrib (Scribble) protein is a cell membrane-associated protein involved in the regulation of the asymmetric distribution of proteins in T cells [24,25]. We then compared the localization of ASP with hScrib in transfected Jurkat cells seeded on polylysine-covered glass slides. As shown in the additional file 2, figure S2, ASP co-localized with endogenous hScrib in membrane projections.

### ASP is expressed in infected Jurkat T cells

**Figure 6:**
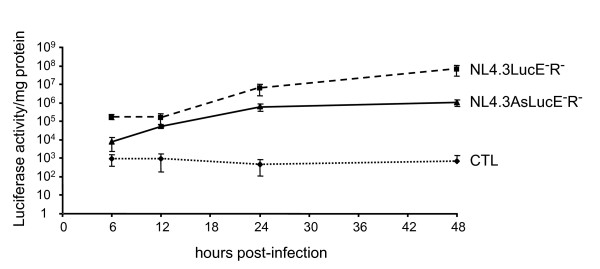
**HIV-1 antisense transcription in infected Jurkat T cells**. Jurkat cells were infected with NL4.3LucE^-^R^- ^or NL4.3AsLucE^-^R^- ^virions pseudotyped with VSVg, and lysed at different time points post-infection and luciferase activity was subsequently measured. Luciferase activities represent the mean value of three measured samples +/- S.D., performed with two different virus preparations for each proviral DNA construct. Luciferase activities are presented on a logarithmic scale. CTL corresponds to levels measured in non-infected cells.

Before analyzing the expression of ASP in infected cells, we first compared 5'-LTR-driven sense transcription with the 3'-LTR antisense transcriptional activity in infected Jurkat cells up to 48 hours post infection (hpi). For this experiment, we made use of previously described proviral DNA constructs containing the luciferase reporter gene inserted in the *nef *coding sequence, either in the sense (pNL4.3LucE^-^R^-^) or antisense direction (pNL4.3AsLucE^-^R^-^) [7,26]. Both molecular proviral clones were separately cotransfected with a VSVg expression vector in 293T cells to produce virions pseudotyped with the VSV envelope. Jurkat cells were subsequently infected with an identical infectious viral titer for both types of virions (MOI = 2). As depicted in Figure 6, at 48 hpi, luciferase activity was notably lower in Jurkat cells infected with NL4.3AsLucE^-^R^- ^virions when compared to cells infected with NL4.3LucE^-^R^- ^virions. Nonetheless, a continuous increase in luciferase activity was observed for both viruses and the 3'-LTR antisense activity was the highest at 48 hpi.

**Figure 7:**
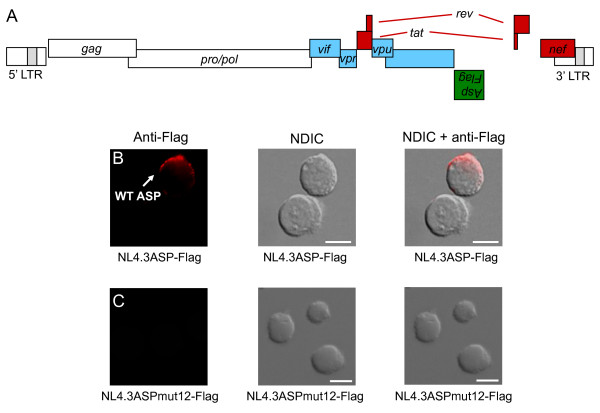
**ASP expression in infected or transfected Jurkat T cells**. (A) Schematic representation of the pNL4.3ASP-Flag vector, which corresponds to a molecular proviral DNA in which ASP was tagged with the Flag epitope at its C-terminal end. Jurkat cells were infected with NL4.3ASP-Flag (B) or NL4.3ASPmut12-Flag (C) virions pseudotyped with VSVg and ASP localization was analyzed as described above. The morphology of the cell was assessed by NDIC. White bars correspond to a scale of 10 μm.

Next, to detect ASP in infected Jurkat cells, we generated the proviral clone, pNL4.3ASP-Flag, in which ASP was tagged with the Flag epitope at its C-terminal end (Figure 7A); the presence of this tag resulted in termination of Env at amino acid 408. Therefore this molecular proviral clone was cotransfected with a VSVg expression vector in 293T cells to produce pseudotyped virions able to infect Jurkat cells. As determined by our analysis on the 3'-LTR transcriptional activity, expression and localization of ASP were analyzed by fluorescence microscopy at the optimal time, i.e. 48 hpi. Although ASP was detected in very few cells, its polarized localization was again confirmed in infected cells (Figure 7B). As negative control, we generated the mutant proviral DNA clone, pNL4.3ASPmut12-Flag, in which the expression of ASP-Flag was inhibited by introducing a stop codon at amino acid 12 of ASP as described above (see Figure 1). No staining was detected in Jurkat cells infected with viruses derived from this mutated construct, although Gag-positive cells were observed as frequently as the other tested proviral DNA (Figure 7C and the additional file 3, figure S3).

Taken together, our results demonstrate for the first time that ASP is detected when expressed from full-length proviral DNA and that its localization is consistent with Jurkat cells overexpressing ASP.

## Discussion

The existence of bidirectional transcription from retrovirus LTRs has been initially suggested based on the identification of conserved ORFs in the antisense strand of their genome, and its demonstration has been mostly focused on human lymphotropic retroviruses. An initial study by Miller [2] had addressed this possibility in HIV-1, and similar ORFs had subsequently been identified on the antisense strand of other retroviruses like HTLV-1 and feline immunodeficiency virus [3,27]. However, the existence of antisense transcription in retroviruses was controversial until the characterization of HBZ in 2002 [8]. Since then, antisense transcription has also been confirmed in HTLV-2 [20] and in gammaretroviruses such as murine leukemia virus [28]. Over the years, transcription initiation has been demonstrated to be a complex process and, in fact, most promoter regions associated to active mammalian genes can transcribe in both sense and antisense directions [29,30]. It seems that retroviruses have developed a mechanism to hijack the bidirectional transcription machinery to produce proteins from sense and antisense transcription. The presence of coding genes can probably stimulate elongation by RNA polymerase II either in the sense direction from the 5' LTR, or in the antisense direction from the 3' LTR. Such a mechanism allows the synthesis of a complex proteome from the proviral genome integrated into the cell chromosome.

Although the synthesis of proteins from antisense transcripts has been clearly demonstrated in the case of HTLV-1 and -2 [8,20], this possibility remains debated for HIV-1. Antisense RNA was however identified in various cell lines chronically infected with HIV-1 [7,23,31]. By RACE analyses, we have recently identified several transcription initiation sites near the 5' border of the 3' LTR and a polyA signal located at 2.4 kb distance from the ASP stop codon [7]. Such transcripts are potentially templates encoding the ASP protein. Indeed, it has been found that translation of the *in vitro*-synthesized antisense RNA yielded a protein with an apparent molecular weight of 19 kDa in SDS-PAGE [23] corresponding to the theoretical molecular weight of ASP. Moreover, this report further described the presence of antibodies against ASP in several sera of HIV-1-infected patients [23]. Interestingly, very recent results support the notion that epitopes derived from antisense transcripts serve as CD8 T-cell targets in HIV-1 infection [21]. Taken together, all these data suggest that the HIV-1 ASP protein should be expressed *in vivo*. However, its detection through Western blot analysis from cellular extracts has not yet been possible. Different reasons can explain the lack of detection of the ASP protein. First of all, antisense retroviral proteins are poorly expressed *in vivo *[8,20]. Levels of antisense transcripts can be 30 to 1000 folds lower than that of sense transcripts [7]. In addition, the negative effect of certain sequences on RNA stability is well known in the case of HIV-1 sense transcripts. For instance, Vpu and Vif proteins are poorly expressed from expression vectors and generation of codon-optimized viral cDNAs can overcome this limitation [32]. Indeed, ASP expression can be improved by codon optimization of its coding sequence (B.B., personal communication). A second concern for the detection of ASP is related to its structure. In this paper, we demonstrate that ASP is localized to the plasma membrane. This localization is consistent with the predicted structure of ASP, which is a highly hydrophobic protein displaying two potential α-helical transmembrane segments. Furthermore, the ASPmut66 protein (deleted of both potential transmembrane domains) did not localize to the plasma membrane, confirming the predicted structure. Its inherent membrane-bound nature makes the characterization of this protein particularly difficult and likely requires special experimental conditions [33]. In addition, characterization of membrane proteins is very difficult because they are usually poorly abundant. All these issues concerning membrane proteins explain the great disparity between current knowledge of soluble versus membrane proteins.

In this paper, by using a strategy different from Western blot analyses, we clearly demonstrate for the first time that ASP is expressed in infected Jurkat T cells. By using fluorescence microscopy, we have first characterized the distribution of ASP tagged with the Flag epitope in Jurkat cells. We then compared ASP localization in these conditions with that in Jurkat cells infected with a proviral clone in which ASP was tagged with the Flag epitope to its C-terminus. We could indeed confirm the polarized localization profile of ASP in infected cells. As expected, this staining pattern was abolished by introducing a stop codon in the *asp *ORF. By using immunoelectron microscopy, expression of ASP has been previously analyzed in HIV-1-infected Sup-T1 cells and the viral protein appeared to concentrate in the nucleus and in the cytoplasm [22]. ASP was also detected in the activated ACH-2 cell line, a chronically infected T cell line. In this study, ASP localized in several cell compartments including the nucleus, the nucleolus, and the mitochondria but not at the plasma membrane [22]. The presence of ASP was detected in the cytoplasm and the nucleus in the vicinity of the cell membranes. However, the nucleolar localization of ASP is unexpected since the nucleolus is a non-membrane bound structure. In our infection experiments, we have never detected ASP associated to the nucleus or the nucleolus. We are unable to explain this discrepancy between the two approaches.

At the moment, the function of ASP remains mysterious. In our studies, we have noted a significant reduction in the level of extracellular p24 production from 293T cells transfected with the mutant pNL4.3ASPmut12 compared to the parental wild-type proviral DNA, but we have been unable to reproduce these results in Jurkat and Sup-T1 cells infected with virions produced with the same mutant. This difference could be explained by the method used to introduce the viral genome into cells. In the case of infection, integration of retroviral DNA into the host genome is an obligatory step for viral protein expression. Depending on the chromosomal location of the integrated provirus, LTR-mediated transcription may vary from 0- to 70-fold. At the moment, we do not know whether ASP is more expressed in transfected 293T cells than in infected Jurkat cells. Similarly, regulation of ASP expression during viral life cycle remains unclear. In the case of HTLV-1, kinetic analysis revealed that antisense transcription was expressed at a low level early after infection and continued to increase before reaching a plateau, showing an inverse correlation between sense/antisense transcription over time [34]. We do not observe a similar trend in the case of HIV-1 but experiments are currently in progress to study the regulation of antisense transcription in primary cells. It is thereby difficult to draw conclusions concerning the function of ASP.

## Conclusion

We demonstrate for the first time that ASP can be produced in infected Jurkat T cells. The *in vivo *detection of ASP gives a novel tool to better understand how HIV-1 is involved in the development of immunodeficiency.

## Methods

### Plasmids and antibodies

The pNL4.3 HIV-1 proviral DNA was obtained from the NIH AIDS Research and Reference Reagent Program (Germantown MD). To produce the pNL4.3-ASPmut12 construct, a NdeI/BamHI fragment containing the *asp *sequence was first cloned in a similarly digested pGL3 basic vector. Using primers 24-8 (5'-GTTGCAACTCACAGTCTGGGGCAT-3') and 24-7 (5'-AGATGCTGTTG**A**GCCTC AATAGCC-3'; the mutated nucleotide is indicated in bold), reverse PCR was used to mutate the cysteine residue in position 12 into a stop codon (TGC into TGA). Sequencing of the entire NdeI/BamHI fragment confirmed the specific mutation after which the fragment was cloned back in the pNL4.3 DNA to replace the wild type segment. The pNL4.3LucE^-^R^- ^vector (containing the luciferase reporter gene and deficient for Env and Vpr synthesis [26]) was generously provided by Dr N.R. Landau. The pNL4.3AsLucE^-^R^- ^vector has previously been described [7]. The Flag-ASP, Flag-ASPΔATG, and Flag-ASPmut66 cDNA fragments were generated by PCR amplification using Deep Vent DNA polymerase and specific sense and antisense primers. The nucleotide sequence coding for the Flag epitope (DYKDDDDK) has been inserted in the sequence of the sense primer. The synthesized cDNA was inserted into the BamHI/EcoRI cloning sites of the linearized pcDNA3ZEO vector. To generate the pNL4.3ASP-Flag construct, the NL4.3-derived NdeI/BamHI fragment cloned in pGL3 basic was used to add NcoI and XbaI sites and displace the stop codon at the 3' end of the ASP ORF by reverse PCR with the following primers: 5'-GCTCTAGATAGAAAAATTCCCCTCCACAATTAAAACTG-3' (sense) and 5'-GTCCATGGCTGTAATTCAACACAACTGTTTAATAGTAC-3' (antisense). Primers permitting the addition of a Flag tag at the COOH end of the ASP ORF, 5'-CATGGGACTACAAGGACGACGACGACAGT-3' (sense) and 5'-CTAGACTTGTCGTCGTCGTCCTTGTAGTCC-3' (antisense), were annealed and inserted in frame at the 3' end of the ASP ORF after NcoI/Xba I digestion. The resulting Flag-tagged ASP ORF was reinserted in the NL4.3 proviral DNA using the NdeI/BamHI sites. The 5'LTR-deleted pNL4.3ASP-Flag Δ5'LTR construct was then generated by NarI digestion and self ligation. pRcActin-lacZ contains the β-galactosidase gene under the control of the human β-actin promoter.

Mouse anti-p24 antibody was purchased from Abcam, while goat anti-mouse IgG was bought from GE Healthcare. Mouse anti-Gag KC57-RD1 antibody was purchased from Beckman-Coulter. We have obtained the mouse anti-Flag antibody from Sigma and the rabbit anti-hScrib (H-300) antibody from Santa Cruz Biotechnology, Inc.

### Transfection, infection, and detection of viral p24 capsid antigen

Transfection experiments in 293T cells were conducted as previously described [35]. Briefly, 293T cells (3 × 10^5^) were plated 24 h prior to transfection in a 6-well plate and were next transfected through the calcium phosphate protocol with 3 μg wild-type or ASP-deficient pNL4.3 proviral DNA construct along with 0.5 μg pRcActin-lacZ (used to normalize transfection experiments). At 48 h post-transfection, supernatants were harvested and levels of the HIV-1 p24 capsid protein was evaluated by a p24-specific ELISA assay as previously described [36]. All experiments were performed in triplicates and final p24 levels are represented as the average of independent triplicate transfection experiments after β-galactosidase normalization. Jurkat and Sup-T1 cells (1 × 10^6 ^cells) were infected with harvested wild-type and ASP-deficient NL4.3 viruses (100 ng p24) in RPMI supplemented with 10% FCS. Extracellular p24 levels were quantified by ELISA at days 3, 7, 10 and 14, and represent the mean values +/- S.D of three independently infected samples.

### Western blot

Transfected 293T cells were washed in PBS and proteins were isolated in a lysis buffer (50 mM, Tris-HCl pH 7.5, 150 mM Nacl, 0.5% NP-40, and protease inhibitor cocktail in tablets). Protein concentrations were then quantified with the BCA protein assay (Thermo Fisher Scientic Inc.). Cellular extracts were migrated on an 8.5% SDS-PAGE and transferred on a PVDF membrane. Membranes were next blocked in 3% BSA and incubated with a polyclonal anti-p24 antibody (1/1000) and were further incubated with horseradish peroxidase-conjugated goat anti-rabbit antibody (1/1000). Signals were detected with the BM chemiluminescence blotting substrate kit (Roche Diagnostics) and membranes were subsequently exposed on an ECL high performance chemiluminescence film (Amersham Biosciences). Densitometric analyses were conducted on three independent Western blot analyses from these transfected cells and mean values +/- S.D. were determined.

### Electron microscopy analysis

Cells were fixed in situ with 2.5% glutaraldehyde in 100 mM cacodylate buffer pH 7.4 for 3 h at 4°C. After three washes in cacodylate buffer, cells were postfixed with 2% osmium tetroxide for 1 h at room temperature and washed in cacodylate buffer. A third fixation in 0.5% tannic acid was performed at room temperature. After extensive washes in 0.1 M Sorensen phosphate buffer pH 7.2, cells were included in a fibrin clot. Cells were then dehydrated and embedded in epony resin (Embed-812; Electron Microscopy Sciences). Sections were counterstained with uranyl acetate and lead citrate alkaline and examined with the transmission electron microscope Hitachi H7100 TEM.

### Fluorescence microscopy analysis

Jurkat cells were cultured in RPMI supplemented with 10% FCS. For transient-transfection assays, cells (10^6^) were transfected with 2 μg plasmids with the device Nucleofector II according to the manufacturer's instructions (Amaxa Biosystems, Lonza). After 48 h, cells were pelleted, washed twice in PBS and overlaid for 30 min at 37°C on glass slides coated or not with polylysine. Cells were then fixed with 4% formalin for 10 min, followed by a treatment of 5 min with NH4Cl 5 mM to remove excess of formaldehyde. Cells were next permeabilized with 0.1% Triton for 7 min. Fixed cells were subsequently incubated with a blocking solution (PBS containing 5% FCS) and then with the primary antibody (mouse anti-Flag M2 antibody; Sigma) for 1.5 h at 37°C. After several washes with PBS, the cells were incubated with the secondary antibody coupled to Alexa Fluor 568 (Invitrogen) for 45 min at room temperature. If necessary the nuclei were stained with Hoechst (Sigma). In certain experiments, cells were stained with fluorescein isothiocyanate-conjugated cholera toxin B (FITC-Co-Tx; Sigma) by incubating the cells with 5 μg/ml of FITC-Co-Tx overnight at 4°C before fixation. Coverslips were mounted with Prolong GOLD (Invitrogen) for direct observation as previously described [25]. Fluorescence images were acquired by a fluorescence microscope (model DC250 Leica), and analysis of the green, red, and blue fluorescence in colocalization experiments was performed with a SP2 Leica confocal microscope.

### Infection and luciferase assays

To produce the pseudotyped HIV-1 particles, 293T cells were plated 16 h before transfection (7 × 10^6 ^cells per flask into six 75 cm^2 ^flasks) and then transfected with the different pNL4.3 proviral DNA (30 μg) and the Vesicular Stomatitis Virus envelope (VSVg) expression vector (18 μg) using the jetPEI™ transfection reagent (Qbiogene) according to manufacturer's instructions. Five hours after transfection, cells were cultured for 48 h in serum-free DMEM. Viruses were then collected by filtering the culture media through a 0.45 μm pore size cellulose acetate membrane and the filtrate was spun down at 100000 g at 4°C during 2 h. After centrifugation, the supernatant was removed and the viral pellet was resuspended in 100-500 μl RPMI supplemented with 5% FCS. Virus stocks were then aliquoted and frozen at -80°C for future use. All virus stocks underwent a single freeze-thaw cycle before use in infection studies. Infectious virus titer was determined by infecting Jurkat cells (10^6^/well in a 24-well plate) with two-fold serial dilution of virus stocks. At 48 hours post-infection (hpi), the percentage of infected cells was determined by intracellular staining of HIV-1 Gag with KC57-RD1 antibodies followed by analysis with an Epics XL flow cytometer (Beckman-Coulter). Jurkat cells (10^6^/well) were infected with an identical multiplicity of infection (MOI = 2) for virus stocks. Luciferase assays were performed in an automated luminometer (Contro XS^3 ^LB960, Berthold technologies) with the Genofax A kit (Yelen, Ensue la Redonne) according to the manufacturer's instructions.

## Competing interests

The authors declare that they have no competing interests.

## Authors' contributions

Contribution: AG, BB, LB, and JMM. designed the research; IC, SL, CT, SL, SB, AV, CAA, and BG. performed research and collected data, BB and JMM analyzed data and wrote the paper. All authors read and approved the final manuscript.

## Supplementary Material

Additional file 1 **Figure S1. Analyses of p24 signals by densitometry**. Densitometric analyses were used to quantify p24 levels and are expressed as a ratio of p24 over GAPDH. Mean values +/- S.D. were calculated from three independent transfection and Western blot analyses. Click here for file

Additional file 2 **Figure S2. ASP colocalizes with hScrib**. Microscopy analysis of endogenous hScrib was performed in Jurkat cells transfected with pcDNA-Flag-ASP already shown in Figure. 5C. Jurkat cells were stained with the rabbit anti-hScrib antibody and goat anti-rabbit immunoglobulin G antibody coupled to FITC while the localization of ASP was analyzed as already described. For localization, analysis of green (anti-hScrib), red (anti-Flag), and merged fluorescence was performed with a confocal microscope. Nuclei were labelled with Hoechst. White bars correspond to a scale of 10 μm. Click here for file

Additional file 3 **Figure S3. Gag expression in Jurkat T cells infected with NL4.3ASPmut12-Flag**. Jurkat cells were infected (A) or not (B) with NL4.3ASPmut12-Flag and infection was confirmed by intracellular staining of HIV-1 Gag with KC57-RD1 antibodies and analyzed as already described. Click here for file
