# Supplementary figures and images for: Polarized expression of the membrane ASP protein derived from HIV-1 antisense transcription in T cells
Source: Retrovirology. 2011 Sep 19;8:74. doi: 10.1186/1742-4690-8-74 (PMC3182985; doi:10.1186/1742-4690-8-74)

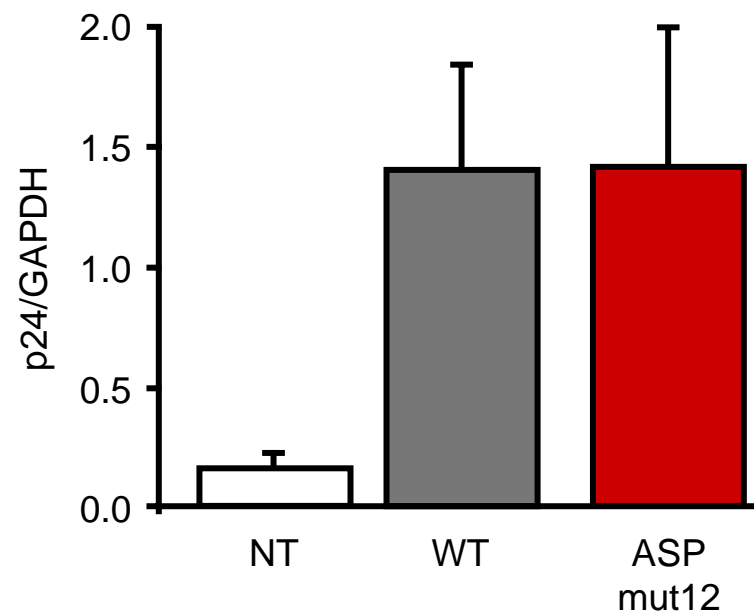

Figure S1

Supplement: Additional file 1 — Figure S1. Analyses of p24 signals by densitometry. Densitometric analyses were used to quantify p24 levels and are expressed as a ratio of p24 over GAPDH. Mean values +/- S.D. were calculated from three independent transfection and Western blot analyses. [file 1742-4690-8-74-S1.PDF]

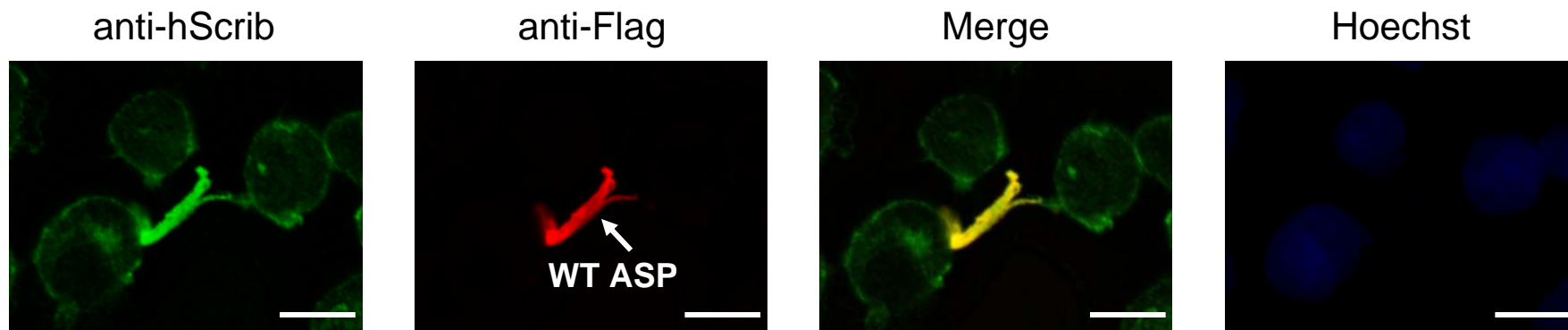

Figure S2

Supplement: Additional file 2 — Figure S2. ASP colocalizes with hScrib. Microscopy analysis of endogenous hScrib was performed in Jurkat cells transfected with pcDNA-Flag-ASP already shown in Figure. 5C. Jurkat cells were stained with the rabbit anti-hScrib antibody and goat anti-rabbit immunoglobulin G antibody coupled to FITC while the localization of ASP was analyzed as already described. For localization, analysis of green (anti-hScrib), red (anti-Flag), and merged fluorescence was performed with a confocal microscope. Nuclei were labelled with Hoechst. White bars correspond to a scale of 10 μm. [file 1742-4690-8-74-S2.PDF]

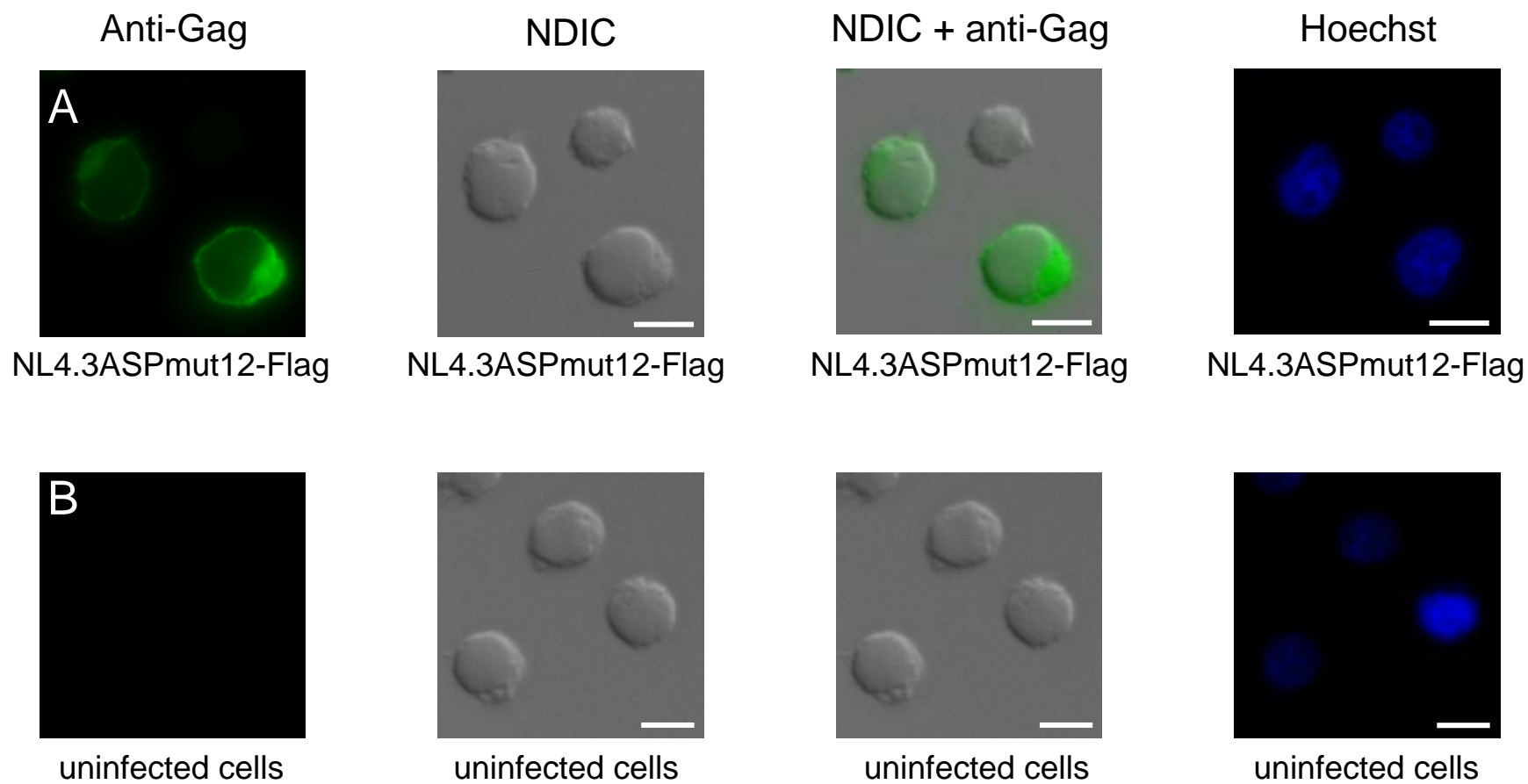

Figure S3

Supplement: Additional file 3 — Figure S3. Gag expression in Jurkat T cells infected with NL4.3ASPmut12-Flag. Jurkat cells were infected (A) or not (B) with NL4.3ASPmut12-Flag and infection was confirmed by intracellular staining of HIV-1 Gag with KC57-RD1 antibodies and analyzed as already described. [file 1742-4690-8-74-S3.PDF]
